# Transferrin‐bearing liposomes entrapping plumbagin for targeted cancer therapy

**DOI:** 10.1002/jin2.56

**Published:** 2019-06-26

**Authors:** Intouch Sakpakdeejaroen, Sukrut Somani, Partha Laskar, Margaret Mullin, Christine Dufès

**Affiliations:** ^1^ Strathclyde Institute of Pharmacy and Biomedical Sciences University of Strathclyde 161 Cathedral Street Glasgow G4 0RE UK; ^2^ College of Medical, Veterinary and Life Sciences University of Glasgow Glasgow G12 8QQ UK

**Keywords:** cancer therapy, liposomes, plumbagin, transferrin, tumour targeting

## Abstract

The therapeutic potential of plumbagin, a naphthoquinone extracted from the officinal leadwort with anticancer properties, is hampered by its failure to specifically reach tumours at a therapeutic concentration after intravenous administration, without secondary effects on normal tissues. Its use in clinic is further limited by its poor aqueous solubility, its spontaneous sublimation, and its rapid elimination *in vivo*. We hypothesize that the entrapment of plumbagin within liposomes grafted with transferrin, whose receptors are overexpressed on many cancer cells, could result in a selective delivery to tumours after intravenous administration. The objectives of this study were therefore to prepare and characterize transferrin‐targeted liposomes entrapping plumbagin and to evaluate their therapeutic efficacy *in vitro* and *in vivo*. The entrapment of plumbagin in transferrin‐bearing liposomes led to an increase in plumbagin uptake by cancer cells and improved antiproliferative efficacy and apoptosis activity in B16‐F10, A431, and T98G cell lines compared with that observed with the drug solution. *In vivo,* the intravenous injection of transferrin‐bearing liposomes entrapping plumbagin led to tumour suppression for 10% of B16‐F10 tumours and tumour regression for a further 10% of the tumours. By contrast, all the tumours treated with plumbagin solution or left untreated were progressive. The animals did not show any signs of toxicity. Transferrin‐bearing liposomes entrapping plumbagin are therefore highly promising therapeutic systems that should be further optimized as a therapeutic tool for cancer treatment.

## Introduction

Cancer, the second leading cause of death in the world, accounted for 9.6 million deaths in 2018 and continues rising worldwide, with an estimation of 16.4 million deaths in 2040 (Bray et al., 2018). Although several therapeutic strategies, such as surgery, radiotherapy, and immunotherapy, can be efficacious against cancers, chemotherapy remains an important treatment for patients diagnosed with cancer (Palumbo et al., 2013).

Among the current anticancer drugs approved from the late 1930s to 2014, approximately 55% were derived from natural sources (Newman & Cragg, 2016). For example, paclitaxel (from the Pacific yew tree), doxorubicin (from *Streptomyces peucetius* bacterium), vincristine (from the periwinkle plant), topotecan (from the *Camptotheca acuminate* tree), and etoposide (from the mayapple plant Podophyllum peltatum) are well‐established drugs available in the market for cancer treatment. In addition, several natural‐derived compounds, such as isoflavones (from soy bean), curcumin oils (from turmeric), and resveratrol (from grape seed) are currently being investigated in clinical trials (Cragg & Pezzuto, 2016). Natural products are therefore an important channel for the discovery of new anticancer agents.

Plumbagin (5‐hydroxy‐2‐methyl‐1,4‐naphthoquinone), a natural‐derived naphthoquinone, isolated from the roots of *Plumbaginaceae* plants (Checker et al., 2018), has been reported to have anticancer effect in various types of cancer, including breast, lung, prostate, cervical, liver, colon, brain, and melanoma cancers (Panichayupakaranant & Ahmad, 2016; Rajalakshmi et al., 2018; Checker et al., 2018). These anticancer effects are mediated through the modulation of cellular redox balance and reduction of glutathione levels, as well as through the activation of apoptotic pathways in cancer cells (Checker et al., 2018). Numerous studies have demonstrated that plumbagin targets several signalling pathways, such as p53, p38, MAPK, STAT3, NF‐κB, FOXM1, MMP2/9, VEGFR2, Ras, Sirtuin1, caspase‐3, JNK, and Wnt/β‐catenin (Sandur et al., 2006; Seshadri et al., 2011; Lai et al., 2012; Niu et al., 2015; Pan et al., 2015; Wang et al., 2015; Zhou et al., 2015; Xue et al., 2016). Plumbagin therefore can be considered as a highly promising agent for cancer therapy, due to its wide spectrum anticancer effects.

However, plumbagin has some limitations that significantly hampered its clinical translation, such as poor solubility in water (79 μg/mL) (Pawar et al., 2016), high lipophilicity (log *P* 3.04) (Pawar et al., 2016), lack of stability (spontaneous sublimation), and low oral bioavailability (less than 40%) (Hsieh et al., 2006). Furthermore, this compound is unable to reach tumours at a therapeutic concentration due to its lack of tumour specificity and rapid elimination (biological half‐life of only 35.89 ± 7.95 min) (Kumar et al., 2011).

To overcome this limitation, we hypothesize that loading plumbagin within liposomes that have the ability to entrap this lipophilic drug would improve its water solubility, prolong its blood circulation time, and sustain its release over a period of time would improve the efficacy of the treatment while reducing the adverse effects of the drug. Several thermosensitive and PEGylated liposomes have been developed to overcome the drawback of plumbagin but with limited efficacy so far (Tiwari et al., 2002; Kumar et al., 2011). They displayed a limited drug loading, were prone to drug leakage, and were unstable. In addition, thermosensitive liposomes could not be translatable to clinical use.

In this study, we developed a new formulation of plumbagin‐loaded liposomes, composed of hydrogenated phosphatidylcholine, cholesterol, distearoyl‐*sn*‐glycero‐3‐phosphoethanolamine, with a mixture of low and high molecular weight PEG (2000 and 5000 Da) at high concentrations to obtain sterically stabilized liposomes. These liposomes will be conjugated to transferrin (Tf), whose receptors are overexpressed on many cancer cells (Daniels et al., 2012). The combination of active targeting transferrin, with the passive accumulation of liposomes in tumours due to the enhanced permeability and retention effect (Maeda, 1992), should provide tumour‐selective targeting of the plumbagin‐loaded liposomes to the cancer cells (Zheng et al., 2010; Guo et al., 2015).

The objectives of this study were therefore (1) to prepare and characterize transferrin‐bearing liposomes entrapping plumbagin, (2) to assess their cellular uptake, antiproliferative, and apoptosis efficacy on cancer cells *in vitro*, and (3) to evaluate their therapeutic efficacy *in vivo*, following intravenous administration to mice bearing tumours.

## Materials and Methods

### Cell lines and reagents

Plumbagin (5‐hydroxy‐2‐methyl‐1,4‐naphthoquinone), human holo‐transferrin (Tf), hydrogenated phosphatidylcholine, cholesterol, and all other chemicals that are not specifically mentioned below were purchased from Sigma Aldrich (Poole, UK). 1,2‐Distearoyl‐*sn*‐glycero‐3‐phosphoethanolamine‐*N*‐(carbonyl‐methoxypolyethyleneglycol 2000), sodium salt (DSPE‐PEG) came from NOF Corporation (Tokyo, Japan). Cholesterol‐PEG5000‐maleimide was obtained from Nanocs (New York, NY).

A431 human epidermoid carcinoma and T98G glioblastoma were purchased from the European Collection of Cell Cultures (Salisbury, UK), while Bioware^®^ B16‐F10‐luc‐G5 mouse melanoma that expresses the firefly luciferase was obtained from Caliper Life Sciences (Hopkinton, MA). Dulbecco's modified Eagle medium and Roswell Park Memorial Institute (RPMI) 1640 cell culture media, foetal bovine serum, l‐glutamine, and penicillin–streptomycin were purchased from Life Technologies (Paisley, UK). Passive lysis buffer was obtained from Promega (Southampton, UK). Vectashield^®^ mounting medium containing 4′,6‐diamidino‐2‐phenylindole (DAPI) was obtained from Vector Laboratories (Peterborough, UK). BD Pharmingen^®^ fluorescein isothiocyanate (FITC) Annexin V Apoptosis Detection Kit I was purchased from BD Biosciences (Franklin Lakes, NJ).

### Preparation and characterization of transferrin‐bearing liposomes entrapping plumbagin

To prepare control liposomes entrapping plumbagin, a mixture of hydrogenated phosphatidylcholine (19.2 mg), DSPE‐PEG (6.4 mg), cholesterol (5.3 mg), and cholesterol‐PEG‐maleimide (1.1 mg) (molar ratios: 60:6:34:0.5) in 3.96 mL PBS (pH 7.4) was shaken at 70°C for 1 h. Plumbagin solution (40 μL, 4 mg, measured from a stock solution of 100 mg/mL prepared in dimethylsulfoxide) was then added to the mixture, followed by probe sonication using Sonics Vibracell^®^ VCX 500 (Sonics^®^, Newtown, CT) for 5 × 2 min.

**Figure 1:**
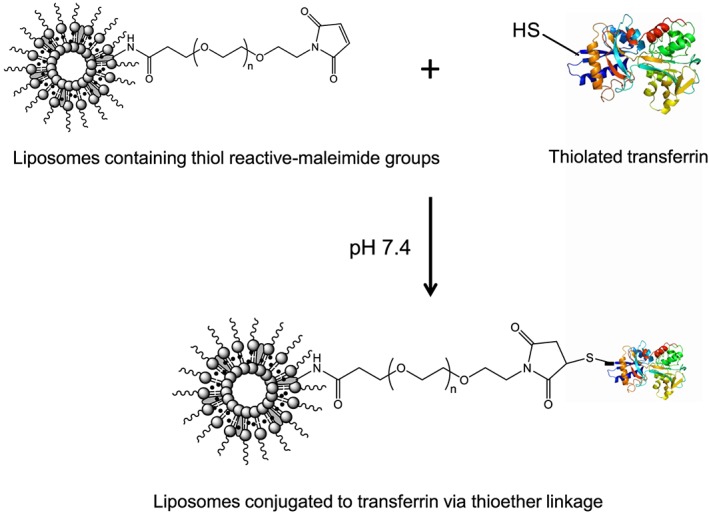
Conjugation of transferrin to the liposomes entrapping plumbagin via thioether linkage.

Transferrin was then thiolated to be able to react with the thiol‐reactive maleimide group of cholesterol‐PEG‐maleimide (Fig. 1). To do so, 10 mg of transferrin were dissolved in 1 mL of 50‐mM sodium phosphate and 150‐mM sodium chloride buffer (pH 8) and reacted with 10‐fold molar excess of 2‐iminothiolane (Traut's reagent, 85 μL, 2 mg/mL in distilled water) at 25°C for 1 h. The thiolated transferrin was then isolated from unreacted Traut's reagent using Vivaspin^®^ six centrifuge tubes with a molecular weight cut‐off of 5 000 Daltons (Sartorius Ltd., Epsom, UK), after centrifugation at 9 500 rpm (10 500 *g*) for 15 min at 20°C (Hermle^®^ Z323K centrifuge, Wehingen, Germany).

The freshly synthesized thiolated Tf was immediately conjugated to the control liposomes under continuous stirring at 25°C for 2 h. Free plumbagin and/or unreacted Tf were removed from both Tf‐bearing and control liposomes using Vivaspin^®^ six centrifuge tubes with a molecular weight cut‐off of 100 000 Daltons (Sartorius Ltd., Epsom, UK) by centrifugation at 7 500 rpm (6 600 *g*) for 15 min at 20°C.

Liposomes were visualized by transmission electron microscopy, using a FEI Tecnai TF20 (ThermoFisher Scientific, Waltham, MA) with a Gatan 794 MultiScan^®^ camera (Gatan, Pleasanton, CA), as previously described (Aldawsari et al., 2011).

The entrapment of plumbagin in the liposomes was quantified by spectrophotometry using an Agilent Varian Cary^®^ 50 UV–Vis spectrophotometer (Agilent Technologies, Santa Clara, CA), following disruption of the liposomes (10 μL) with isopropanol (990 μL). Absorbance of the drug was measured at a λ_max_ of 420 nm.

The grafting of transferrin to the liposomes was quantified by Lowry assay, as previously reported (Dufès et al., 2000).

Size and zeta potential of the liposomes were respectively determined by photon correlation spectroscopy and laser Doppler electrophoresis using a Zetasizer Nano‐ZS^®^ (Malvern Instruments, Malvern, UK).

### Stability of the liposomes

The stability of the liposomes was assessed using three different batches of liposomes. All samples were placed in vials protected from light and were kept at 4°C for 4 weeks. The size and zeta potential of the samples were respectively measured by photon correlation spectroscopy and laser Doppler electrophoresis at specific time points (on Days 0, 7, 14, 21, and 28). The amount of plumbagin remaining in the liposomes was quantified by spectrophotometry compared with the initially entrapped amount.

### Drug release from the liposomes

To confirm that plumbagin could be released from the liposomes, the release profile of the drug was determined by dialysis at pH 7.4. Plumbagin formulated as Tf‐bearing, control liposomes, or in solution (500 μg in phosphate buffer) was placed into a SnakeSkin^®^ dialysis tube with a molecular weight cut‐off of 3 500 Daltons (ThermoFisher Scientific) and was dialyzed against 50 mL of phosphate buffer (pH 7.4) at 37°C under stirring; 1‐mL sample of the dialysate was withdrawn in triplicates at specific time points (30 min, then every hour for the first six hours (1, 2, 3, 4, 5, and 6 h), then every 2 h for the next 6 h (8, 10, and 12 h), and every 24 h for 3 days) and replaced with an equal volume of fresh medium. The amount of plumbagin in the samples was quantified by spectrophotometry and reported as a percentage cumulative release.

### *In vitro* biological characterization

#### Cell culture

B16‐F10‐luc‐G5, A431, and T98G cell lines were grown in either RPMI‐1640 medium (for B16‐F10‐luc‐G5 cells) or in Dulbecco's modified Eagle medium (for A431 and T98G cells) supplemented with 10% (v/v) foetal bovine serum, 1% (v/v) l‐glutamine, and 0.5% (v/v) penicillin–streptomycin. Cells were cultured at 37°C in a humid atmosphere of 5% carbon dioxide.

#### Cellular uptake

Quantification of cellular uptake of plumbagin formulated as Tf‐bearing, control liposomes, or free in solution was carried out by spectrophotometry and flow cytometry.

Cells were seeded at a density of 2 × 10^5^ cells per well in 6‐well plates and grown at 37°C for 72 h before being treated with plumbagin (10 μg per well), either entrapped in transferrin‐bearing liposomes, control liposomes, or in solution. After 3 h treatment, the cells were harvested and washed twice with cold phosphate buffered saline (PBS) (3 mL). Cells were then lysed with 5% Triton‐X (1 mL per well) and incubated for another 24 h at 37°C. Cell lysates were then centrifuged at 10 000 rpm (9 300 *g*) for 15 min using an IEC Micromax^®^ centrifuge (ThermoFisher Scientific). The amount of plumbagin in the surfactant was quantified by spectrophotometry (λ_max_: 420 nm), using a FlexStation 3^®^ multi‐mode microplate reader (Molecular Devices, Sunnyvale, CA).

To further confirm the advantage of using transferrin‐bearing liposomes for improving the cellular uptake, plumbagin was replaced with coumarin‐6 as a fluorescent lipophilic drug model for quantitative and qualitative measurement of drug cellular uptake in B16‐F10‐luc‐G5 cells.

For flow cytometry quantification, the cells were seeded and grown as previously described before being treated with coumarin‐6 (50 ng per well), either entrapped in transferrin‐bearing liposomes, control liposomes, or in solution. After 2‐h incubation, the cells were washed twice with cold PBS (3 mL) and harvested using TrypLE^®^ Express (250 μL). Subsequently, 500 μL RPMI‐1640 medium was added to the cell suspension. Mean fluorescence intensity (MFI) of coumarin‐6 taken up by the cells was quantified by flow cytometry using a FACSCanto^®^ flow cytometry (BD Biosciences), with a FITC filter (Exc_max_ = 494 nm/Em_max_ 520 nm). Ten thousand cells (gated events) were counted for each sample.

The cellular uptake of coumarin‐6 was qualitatively assessed using confocal microscopy. B16‐F10‐luc‐G5 cells (1 × 10^5^ cells per well) were seeded on coverslips in 6‐well plates and were grown for 24 h at 37°C. They were treated with coumarin‐6 (1 μg per well), either entrapped in Tf‐bearing liposomes, control liposomes, or in solution. After 2 h, the medium was removed, and cells were washed twice with cold PBS (3 mL) before being fixed with 2 mL formaldehyde solution (3.7% in PBS) for 10 min at 25°C. They were then washed twice with PBS (3 mL) and incubated at 25°C with 3 mL Triton‐X100 solution (0.1%) for 5 min, before a further incubation with 3 mL bovine serum albumin (1% w/v in PBS) for 30 min at 37°C to reduce the nonspecific binding.

Cells were then stained with Alexa Fluor^®^ 647 dye (one unit of dye diluted in 200 μL of PBS), incubated for 20 min at 25°C, before a final wash with 3 mL PBS. Upon staining of the nuclei with Vectashield^®^ mounting medium containing DAPI, the cells were examined using a Leica TCS SP5 confocal microscope (Wetzlar, Germany). DAPI (staining the cell nuclei) was excited with the 405 nm laser line (emission bandwidth: 415–491 nm), while Alexa Fluor^®^ 647 (which stained the cell cytoplasm) was excited with the 633 nm laser line (emission bandwidth: 645–710 nm), and coumarin‐6 was excited with the 505‐nm laser line (emission bandwidth: 515–558 nm).

### Mechanisms of cellular uptake

The mechanisms involved in the cellular uptake of coumarin‐6 entrapped in Tf‐bearing liposomes were investigated using various uptake inhibitors. B16‐F10‐luc‐G5 cells were seeded in 6‐well plates at a density of 2 × 10^5^ cells per well and incubated for 24 h. Cells were then preincubated with chlorpromazine (20 μg/mL), filipin (4 μg/mL), and colchicine (40 μg/mL) at 37°C. After 30 min incubation, treatment was removed and replaced with fresh medium containing 50 ng/mL of coumarin‐6 (either entrapped in Tf‐bearing, control liposomes, or in solution) and the same concentration of each inhibitor (except chlorpromazine, added at a concentration of 5 μg/mL) for a further 2 h incubation at 37°C. Cells were then washed and processed for flow cytometry analysis as previously described.

### *In vitro* antiproliferative activity

The antiproliferative activity of plumbagin entrapped in transferrin‐bearing liposomes was assessed using a standard 3‐(4,5‐dimethylthiazol‐2‐yl)‐2,5‐diphenyl‐tetrazolium bromide assay. B16‐F10‐luc‐G5, A431, and T98G cells were seeded at a density of 5 000 cells per well in 96‐well plates and grown for 24 h before treatment. They were then treated with plumbagin entrapped in Tf‐bearing liposomes, control liposomes, or in solution, at final drug concentrations ranging from 7.81 × 10^−3^ to 10 μg plumbagin/mL). Following 24‐h treatment, the antiproliferative activity of the formulations was evaluated by measurement of the growth inhibitory concentration for 50% of the cell population (IC_50_) in a standard 3‐(4,5‐dimethylthiazol‐2‐yl)‐2,5‐diphenyl‐tetrazolium bromide (MTT) assay (*n* = 15).

#### Cell apoptosis assay

The number of apoptotic cells following treatment with plumbagin entrapped in transferrin‐bearing liposomes was determined using BD Pharmingen^®^ FITC Annexin V apoptosis detection kit (BD Biosciences), as described in the manufacturer's instructions. Cells were seeded in 6‐well plates at a density of 2 × 10^5^ cells per well and grown for 24 h before being treated with plumbagin (1 μg per well) entrapped in Tf‐bearing liposomes, control liposomes, or in solution. After 4 h treatment, the cells were harvested and centrifuged at 2000 rpm (370 *g*) for 5 min using an IEC Micromax^®^ centrifuge (ThermoFisher Scientific). Subsequently, the cell pellets were resuspended in 200 μL 1× Annexin V Binding Buffer (10× of the buffer containing 0.1‐M Hepes/NaOH (pH 7.4), 1.4‐M NaCl, and 25‐mM CaCl_2_). Cell suspension (100 μL) was then transferred to a 5‐mL culture tube, followed by 5 μL of Annexin V‐FITC labelling reagent and 5 μL of propidium iodide and incubated for 15 min at 20°C protected from light. After incubation, 400 μL of Annexin V Binding Buffer was added to each tube before analysis of apoptosis using a FACSCanto^®^ flow cytometer (BD Biosciences). Ten thousand cells (gated events) were counted for each sample. The results were reported as percentages of specific cell populations (live cells, cells in early apoptosis, late apoptosis, and necrosis).

### In vivo tumouricidal activity

The *in vivo* experiments were approved by the local ethics committee (the University of Strathclyde Animal Welfare and Ethical Review Body), complied with the ARRIVE guidelines and were carried out in accordance with the UK Home Office regulations (UK Animals (Scientific Procedures) Act 1986).

B16‐F10‐luc‐G5 cancer cells in exponential growth were subcutaneously implanted to both flanks of female immunodeficient BALB/c mice (1 × 10^6^ cells per flank). When tumours were palpable and reached a diameter of 5 mm, the animals were randomized into groups of five. They were treated with plumbagin entrapped in Tf‐bearing liposomes, control liposomes, or in solution, by intravenous tail vein injection (2 mg/kg of body weight per injection) once every 2 days for 10 days. The weight of the animal was measured daily as a surrogate marker of the toxicity of the treatments. The tumour volume was determined by calliper measurements (volume = d^3^ × π/6). The results were expressed as relative tumour volume (rel. Volt_x_ = Volt_x_/Volt_0_), and the responses were classified analogous to Response Evaluation Criteria in Solid Tumors guidelines (Eisenauer et al., 2009). Progressive disease was defined as an increase in relative tumour volume higher than 1.2‐fold, stable disease as a relative volume between 0.7 and 1.2 of starting volume, partial response as measurable tumour with a volume reduction of more than 30% (0–0.7), and complete response as the absence of any tumour.

The therapeutic efficacy of these treatments was also assessed by bioluminescence imaging, using an IVIS Spectrum (Caliper Life Sciences). Mice bearing subcutaneous B16F10‐luc‐G5 tumours were intravenously injected with treatments as described above. On alternate days (Days 1, 3, 5, 7, and 9), they were intraperitoneally injected with the luciferase substrate d‐luciferin (150 mg/kg body weight), then anesthetized using isoflurane inhalation 10 min before imaging. The light emitted from the bioluminescent tumours was detected for 2 min using Living Image^®^ software (PerkinElmer, Waltham, MA) and displayed as a pseudo‐colour overlay onto a greyscale image of the animal. Identical illumination settings were used for acquiring all images.

### Statistical analysis

Results were expressed as means ± standard error of the mean. Statistical significance was assessed by one‐way analysis of variance and Tukey's multiple comparison posttest (Minitab^®^ software, State College, PE). Differences were considered statistically significant for *P* values lower than 0.05.

## Results

### Preparation and characterization of transferrin‐bearing liposomes entrapping plumbagin

**Figure 2:**
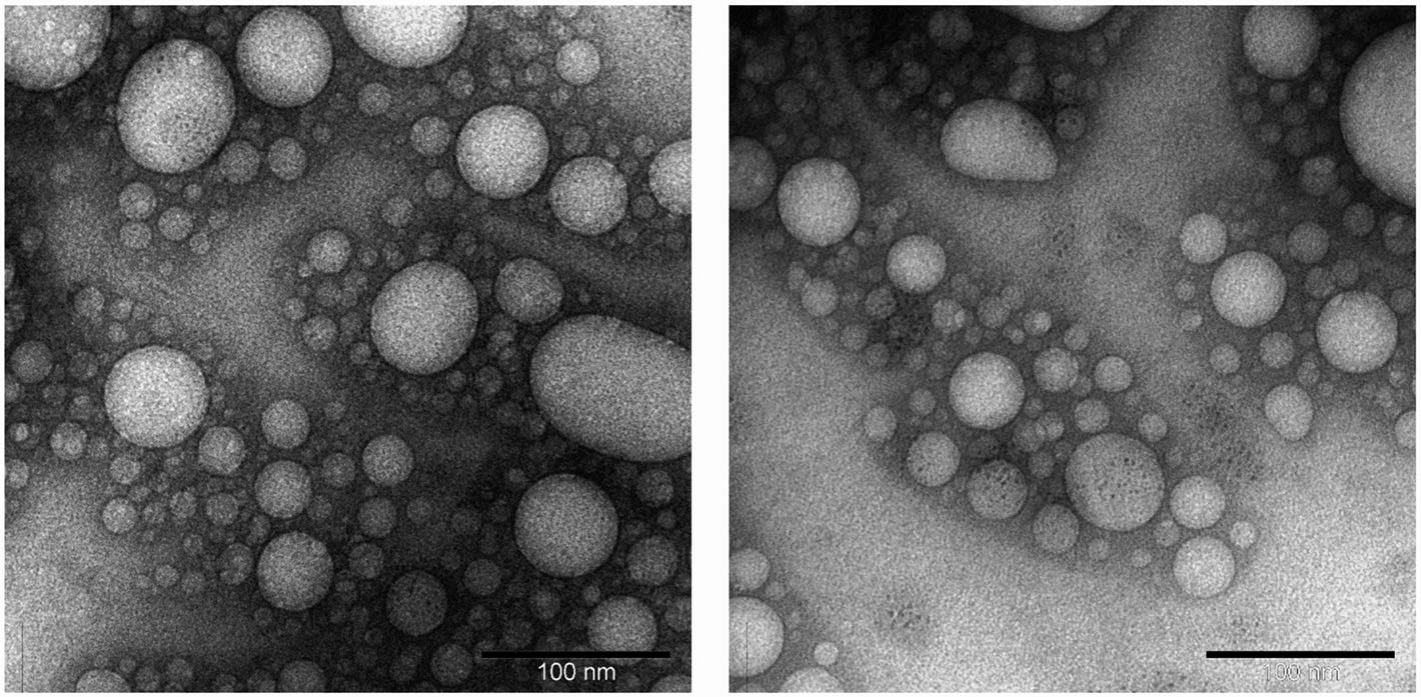
Transmission electron micrograph pictures of Tf‐bearing (left) and control (right) unilamellar liposomes entrapping plumbagin (Bar: 100 nm).

Transferrin‐bearing and control unilamellar liposomes entrapping plumbagin have been successfully prepared by probe sonication, as confirmed by transmission electron microscopy imaging (Fig. 2). The percentage of drug loading within these liposomes was relatively high, respectively 79.2 ± 0.3% for transferrin‐bearing liposomes and 78.4 ± 0.4% for control liposomes. The amount of transferrin conjugated to the liposomes was 5.1 ± 0.1 mg (50.7 ± 0.5% of the initial transferrin added). As expected, the conjugation of transferrin to the surface of the liposomes resulted in a larger average size of 113 ± 2 nm (polydispersity: 0.33 ± 0.01) than that of control liposomes (106 ± 1 nm, polydispersity: 0.32 ± 0.01). It slightly decreased their zeta potential compared with that observed for control liposomes (−18.4 ± 0.4 mV and −17.2 ± 0.1 mV, respectively, for transferrin‐bearing and control liposomes).

### Stability of the liposomes

**Figure 3:**
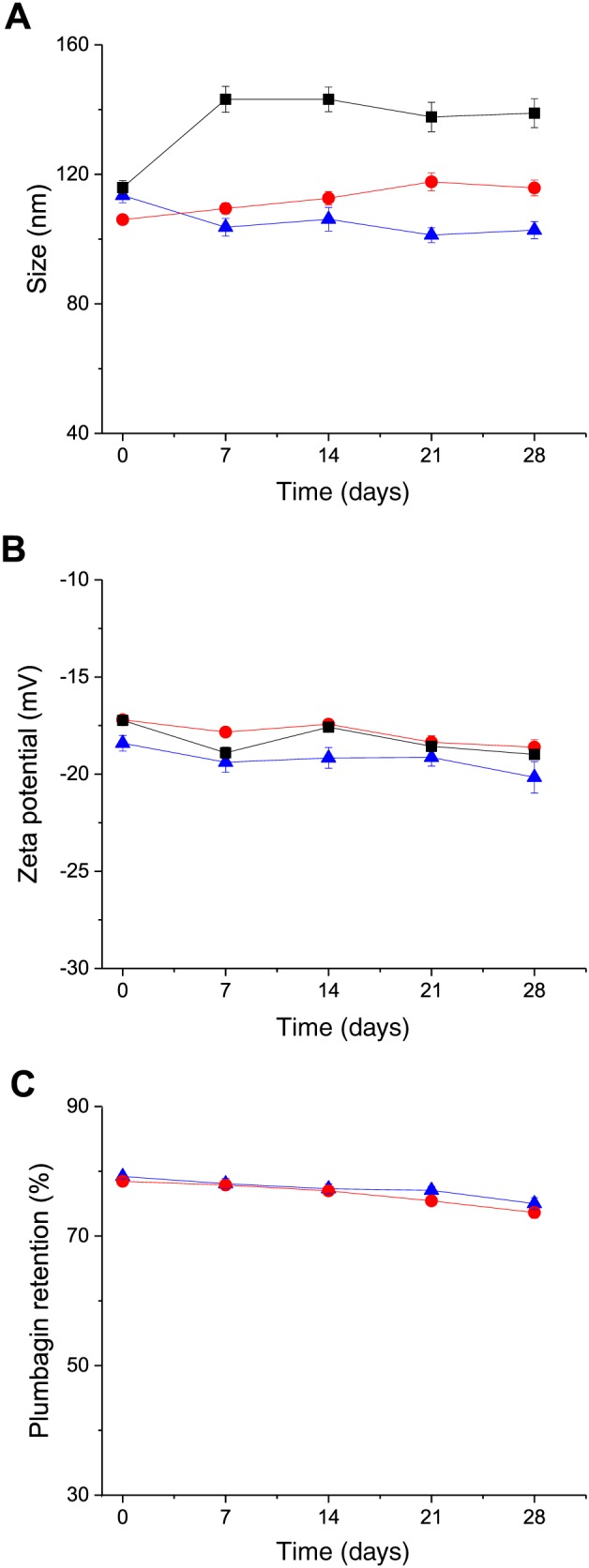
Size (A), zeta potential (B), and percentage retention (C) of plumbagin in Tf‐bearing (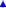, blue) and control liposomes (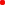, red) (▪, black: blank liposomes) after storage at 4°C for 4 weeks (n = 3).

Transferrin‐bearing liposomes were found to be stable when stored at 4°C for at least 4 weeks (Fig. 3). They displayed a slight decrease in size within 28 days (from 113 ± 2 nm at Day 0 to 102 ± 2 nm at Day 28), unlike control liposomes, whose size slightly increased (from 106 ± 1 nm at Day 0 to 115 ± 2 nm at Day 28). However, blank liposomes appeared to be less stable and significantly increased in size during the experiment (from 115 ± 2 nm at Day 0 to 138 ± 4 nm at Day 28) (Fig. 3A).

The zeta potential of the three formulations remained stable for 28 days (−18.4 ± 0.4 mV at Day 0 to −20.2 ± 0.8 mV at Day 28 for Tf‐bearing liposomes, −17.2 ± 0.1 mV at Day 0 to −18.6 ± 0.4 mV at Day 28 for control liposomes, and −17.2 ± 0.2 mV at Day 0 to −19.0 ± 0.2 mV at Day 28 for blank liposomes) (Fig. 3B).

In terms of drug leakage, the percentage of plumbagin retention in both transferrin‐bearing and control liposomes remained stable, with a slight decrease of plumbagin (less than 5%) over 4 weeks (from 79.1 ± 0.4% to 75.0 ± 0.9% for transferrin‐bearing liposomes and from 78.5 ± 0.4% to 73.6 ± 0.9% for control liposomes) (Fig. 3C).

### Drug release from the liposomes

**Figure 4:**
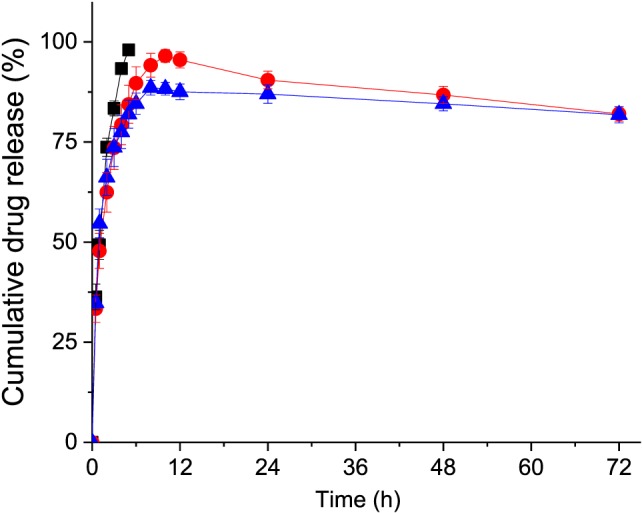
*In vitro* drug release profile of plumbagin formulated as Tf‐bearing liposomes (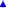, blue), control liposomes (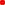, red), or as free drug in solution (▪, black) in phosphate buffer at pH 7.4 over 72 h (n = 3).

Transferrin‐bearing and control liposomes showed similar sustained release profile of plumbagin at pH 7.4, while plumbagin in solution diffused through the dialysis membrane to be completely released within 4 h (Fig. 4). The conjugation of transferrin to the surface of liposomes had a slight impact on the release profile of plumbagin, with a percentage cumulative release of 88.3 ± 1.5%, slightly lower than that observed with control liposomes (96.5 ± 1.5% during the first 10 h).

### Cellular uptake

**Figure 5:**
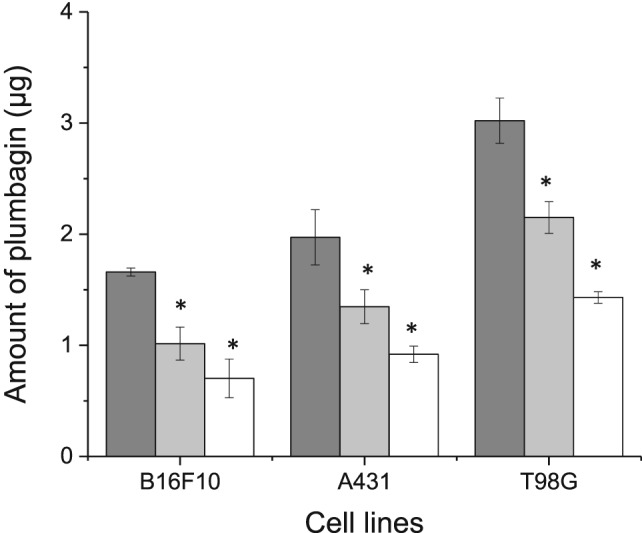
Cellular uptake of plumbagin (10 μg per well) either formulated as Tf‐bearing liposomes (dark grey), control liposomes (grey), or as free drug in solution (white), in B16‐F10, A431, and T98G cell lines (n = 5) (^*^ P < 0.05 vs. Tf‐bearing liposomes).

The entrapment of plumbagin in transferrin‐bearing liposomes significantly improved plumbagin uptake by the three tested cell lines in comparison with control liposomes (by at least 1.4‐fold) and plumbagin solution (by at least 2‐fold) (1.66 ± 0.04 mg, 2.16 ± 0.18 mg, and 3.02 ± 0.20 mg following treatment with transferrin‐bearing liposomes respectively in B16‐F10, A431, and T98G cells; 0.70 ± 0.17 mg, 0.92 ± 0.07 mg, and 1.43 ± 0.05 mg following treatment with plumbagin solution respectively in B16‐F10, A431, and T98G cells) (Fig. 5). The highest intracellular amount of plumbagin was found in T98G cells incubated with transferrin‐bearing liposomes, which was significantly higher than that observed after treatment with control liposomes and free plumbagin, respectively, by 1.4‐fold and 2.1‐fold.

**Figure 6:**
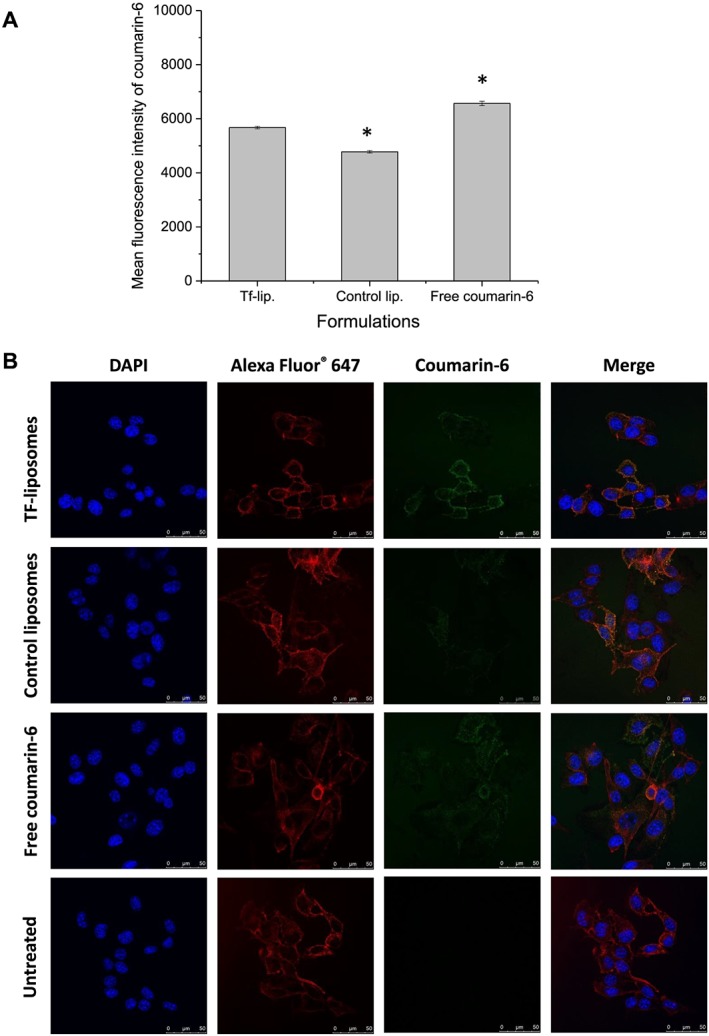
Cellular uptake of coumarin‐6 entrapped in Tf‐bearing liposomes, control liposomes, or as solution in B16‐F10 cells: (A) Mean fluorescence intensity of coumarin‐6 in the cells (n = 3) and (B) qualitative analysis by confocal microscopy.

These results were comparable with the cellular uptake of liposomes entrapping coumarin‐6 as a fluorescent lipophilic drug model in B16‐F10 cells (Fig. 6A). The conjugation of transferrin to the liposomes significantly increased coumarin‐6 uptake (MFI of 5673 ± 49 a.u.) compared with control liposomes (MFI of 4779 ± 48 a.u.). However, the highest uptake was observed following treatment with coumarin‐6 solution (MFI of 6567 ± 79 a.u.), which might occur by passive diffusion.

The cellular uptake of coumarin‐6 was also qualitatively confirmed by confocal microscopy (Fig. 6B). As expected, Tf‐bearing liposomes led to a higher cellular uptake of coumarin‐6 compared with that observed in control liposomes. Cells treated with free plumbagin solution showed plumbagin‐derived fluorescence in the cytoplasm, probably due to the nonspecific diffusion of the drug. Coumarin‐6‐derived fluorescence was disseminated in the cytoplasm following all treatments, with no visible colocalization within the nucleus after 2‐h incubation with the treatments.

### Mechanisms of cellular uptake

Chlorpromazine, filipin, and colchicine were used to inhibit clathrin‐mediated, caveolae‐mediated, and macropinocytosis‐mediated endocytosis, respectively (Cheng et al., 2014).

**Figure 7:**
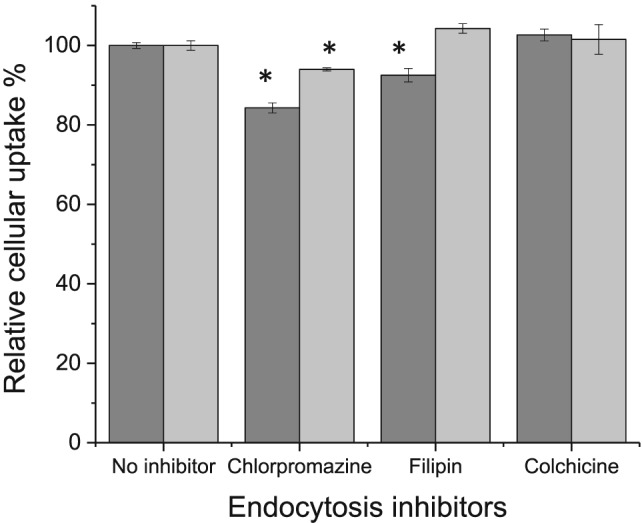
Relative cellular uptake of coumarin‐6 entrapped in Tf‐bearing liposomes (dark grey) or control liposomes (light grey), in the presence of endocytosis inhibitors, in B16‐F10 cells (n = 3).

Pretreatment of B16‐F10 cells with chlorpromazine significantly decreased the cellular uptake of coumarin‐6 entrapped in Tf‐bearing liposomes, which was 16% lower than that observed without pretreatment and 9.7% lower than that observed with control liposomes (respectively 84.3 ± 1.7% and 94.0 ± 0.4% cellular uptake following treatment with Tf‐bearing and control liposomes, with the relative cellular uptake without inhibitor set at 100%) (Fig. 7). The cellular uptake of coumarin‐6 entrapped in Tf‐bearing liposomes was also partially inhibited by filipin, unlike control liposomes. It decreased to 92.5 ± 1.7% compared with that measured in cells without pretreatment. Colchicine, however, did not inhibit the cellular uptake of Tf‐bearing and control liposomes, meaning that macropinocytosis‐mediated endocytosis pathway was not involved in the cellular internalization of these liposomes.

### *In vitro* antiproliferative activity

**Figure 8:**
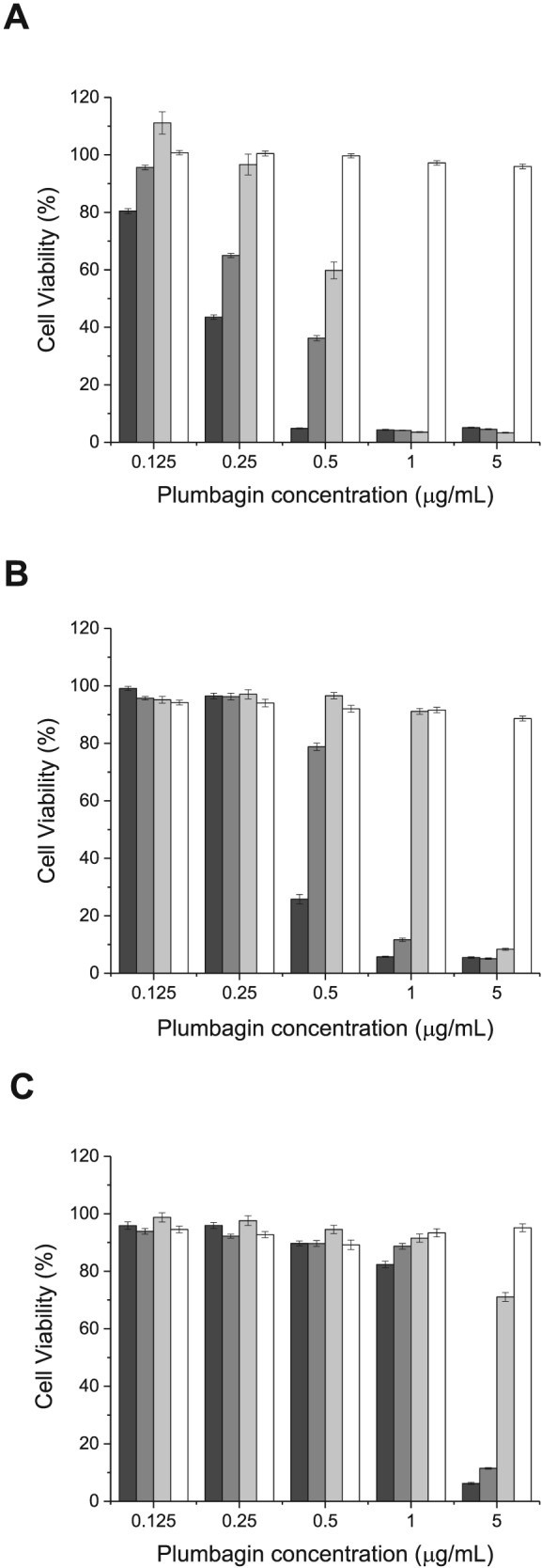
Cytotoxicity of plumbagin entrapped in transferrin‐bearing liposomes (black), control liposomes (dark grey), or free in solution (light grey), on B16‐F10 (top), A431 (middle), and T98G cells (bottom), following 24‐h treatment (control: blank liposomes (white)) (n = 15).

The entrapment of plumbagin in liposome formulations significantly improved the antiproliferative activity of plumbagin, compared with the free solution, by at least 1.5‐fold (Table 1, Fig. 8). The conjugation of transferrin to the liposomes further increased plumbagin antiproliferative efficacy, by 2.3‐fold for B16‐F10 cells, 4.3‐fold for A431 cells, and 4.2‐fold for T98G cells, compared with that of plumbagin solution following 24‐h treatment. The IC_50_ could not be determined following treatment of the cells with blank liposomes.

**Table 1:** Anti‐proliferative efficacy of plumbagin entrapped in transferrin‐bearing liposomes, control liposomes, or as free drug in solution, expressed as IC_50_ values, in B16‐F10, A431, and T98G cells, following 24 h treatment (n = 15).

Cell lines	IC_50_ (μg/mL) (mean ± SEM)			
	Tf‐bearing liposomes	Control liposomes	Plumbagin solution	Blank liposomes
B16‐F10	0.22 ± 0.01	0.31 ± 0.03	0.51 ± 0.02	n.d.
A431	0.41 ± 0.01	0.63 ± 0.02	1.78 ± 0.19	n.d.
T98G	1.47 ± 0.27	2.04 ± 0.35	6.19 ± 0.19	n.d.

n.d., not determined; SEM, standard error of the mean.

Plumbagin loaded in transferrin‐bearing liposomes exhibited the highest antiproliferative efficacy against B16‐F10 cells (IC_50_: 0.22 ± 0.01 μg/mL), followed by A431 cells (IC_50_: 0.41 ± 0.01 μg/mL). However, transferrin‐bearing liposomes entrapping plumbagin only exerted a limited antiproliferative effect in T98G cells (IC_50_: 1.47 ± 0.27 μg/mL).

### Cell apoptosis assay

**Figure 9:**
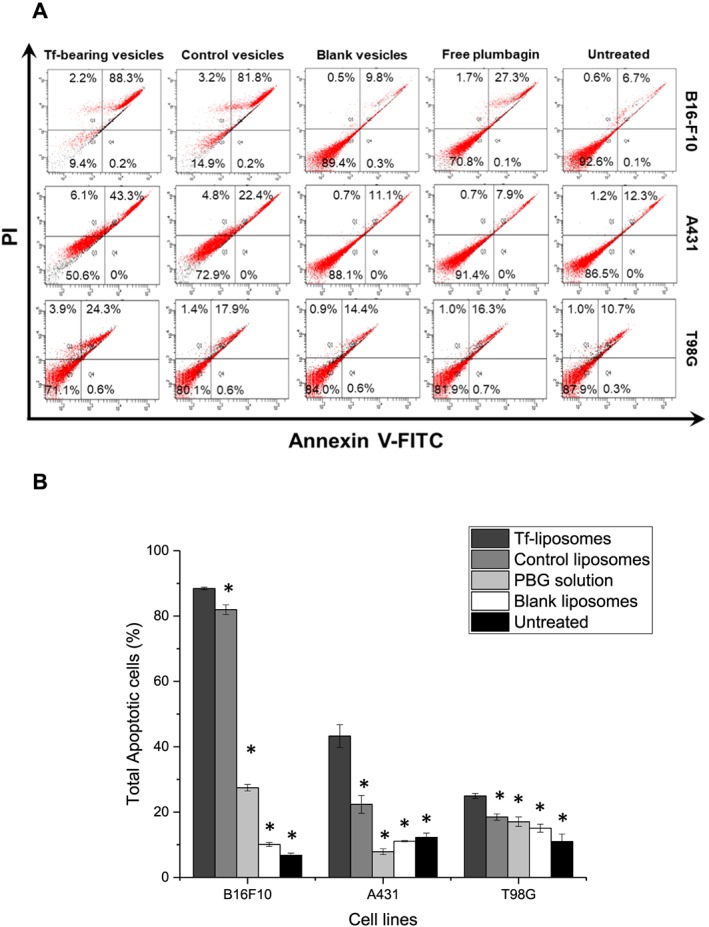
Apoptosis induction of B16‐F10, A431, and T98G cells treated with plumbagin (1 μg) entrapped in Tf‐bearing liposomes, control liposomes, or free in solution after 4‐h treatment. (A) Flow cytometric plots showing the percentage of specific cell populations (live, early apoptosis, late apoptosis, and necrosis). (B) Percentage of total apoptotic cells (n = 3) (*P < 0.05 vs. Tf‐bearing liposomes).

The Tf‐bearing liposomes entrapping plumbagin (1 μg/mL, 5.3 μM) significantly led to a higher cellular apoptosis in B16‐F10 cells compared with that of control liposomes and free plumbagin, with 88.4 ± 0.4% of cells being apoptotic following treatment with Tf‐bearing liposomes, compared with 82.0 ± 1.5% apoptotic cells following treatment with control liposomes. By contrast, only 27.5 ± 1.0% of cells were apoptotic when treated with free plumbagin (Fig. 9).

In A431 cells, the apoptosis effect of Tf‐bearing liposomes (total apoptosis of 43.3 ± 3.5% cells) was lower than what observed with B16‐F10 cells but was still 1.9‐fold higher than that observed following treatment with control liposomes (total apoptosis of 22.4 ± 3.5% cells). Free plumbagin only exerted a very limited apoptosis effect on this cell line at the tested conditions (7.9 ± 0.9% apoptotic cells following treatment with free plumbagin).

In T98G cells, the apoptotic effect of TF‐bearing liposomes was further reduced compared with that of the two other cell lines but was still statistically higher than that observed following treatment with control liposomes and free drug in solution (total apoptosis of 24.9 ± 0.8% cells following treatment with Tf‐bearing liposomes, 18.5 ± 1.0% cells for control liposomes, and 17.1 ± 1.5% cells for free plumbagin).

### *In vivo* tumouricidal activity

**Figure 10:**
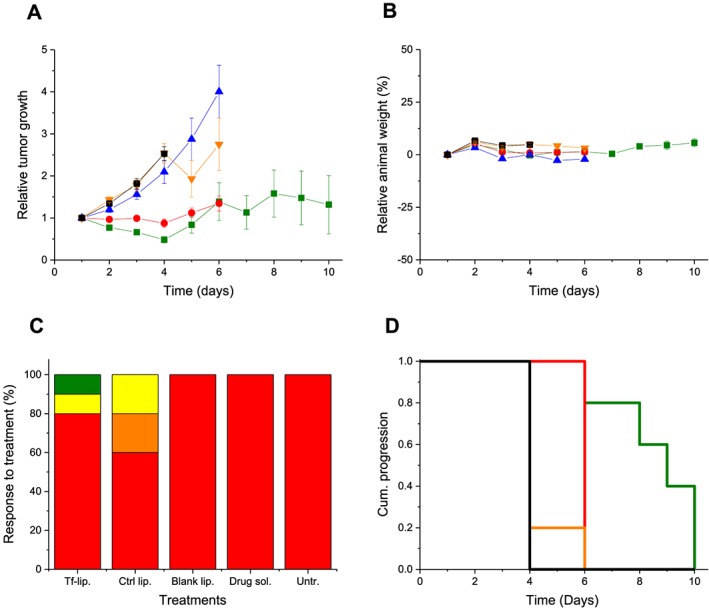
(A) Tumour growth studies in a B16‐F10 murine model after intravenous administration of transferrin‐bearing liposomes entrapping plumbagin (2 mg/kg of body weight/injection) (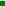, dark green), control liposomes entrapping plumbagin (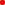, red), bank liposomes (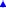, blue), plumbagin solution (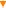, orange), untreated tumours (▪, black) (n = 10). (B) Variations of the animal body weight throughout the treatment (colour coding as in A). (C) Overall tumour response to treatments at the end of the study, classified in accordance with the Response Evaluation Criteria in Solid Tumors (Eisenauer et al., 2009) (red: progressive response; orange: stable response; yellow: partial response; and green: complete response). (D) Time to disease progression. The Y axis gives the proportion of surviving animals over time. Animals were removed from the study once their tumour reached 10‐mm diameter (color coding as in A).

The intravenous injection of plumbagin entrapped in Tf‐bearing liposomes and control liposomes led to a high variability of response to treatment within the same group of mice and an overall reduced tumour growth compared with plumbagin solution treatment (Fig. 10A). For these two treatments, some tumours kept regressing, while others started growing.

At Day 6, the mice bearing growing tumours had to be euthanized due to their tumours reaching the maximum allowed size. The remaining mice whose tumours were regressing or had completely disappeared were kept until the end of the study (Day 10). By contrast, tumours treated with plumbagin solution or blank liposomes grew steadily at a growth rate close to that observed for untreated tumours.

**Figure 11:**
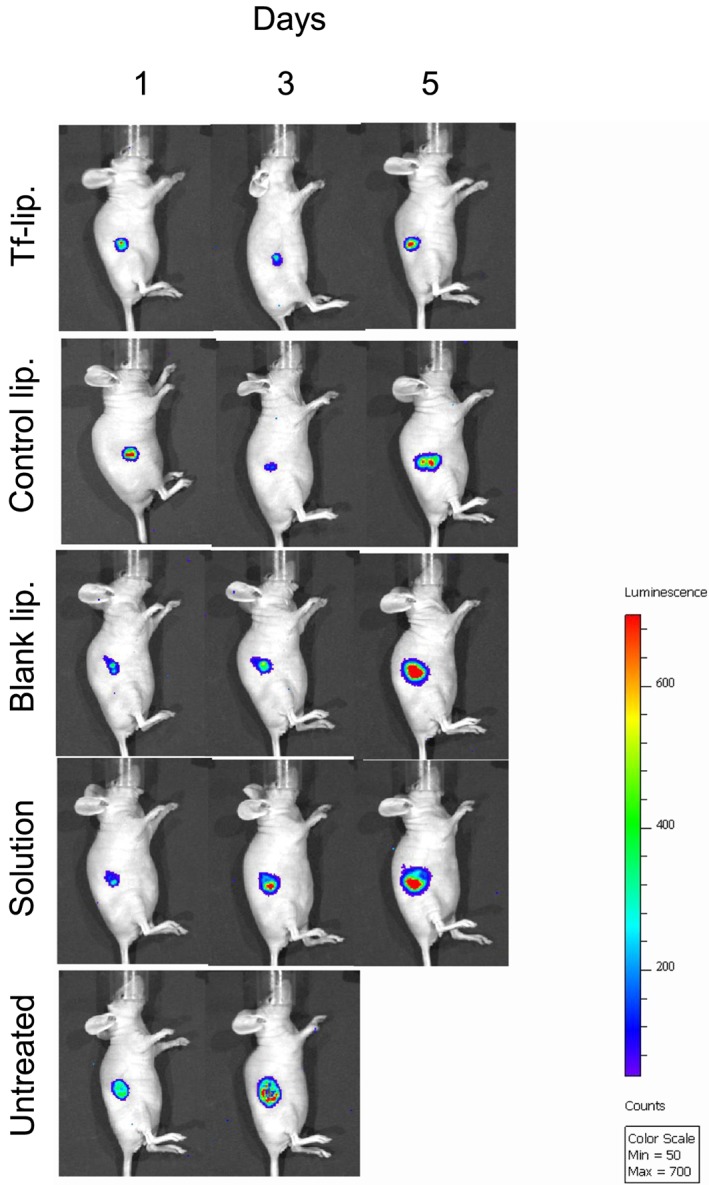
Bioluminescence imaging of the tumouricidal activity of plumbagin entrapped in F‐bearing liposomes(“Tf‐lip.”), control liposomes (“control lip.”), or as solution (“Solution”) in a B16‐F10‐luc tumour model (Controls: Blank liposomes: “Blank lip.” and untreated tumours “Untreated”). The scale indicates surface radiance (photons/s/cm^2^/steradian).

The therapeutic effect resulting from treatment with liposomes entrapping plumbagin was also qualitatively confirmed by bioluminescence imaging on mice bearing subcutaneous B16‐F10‐luc tumours (Fig. 11). Luciferase expression in the tumours treated with the Tf‐bearing and control formulations decreased from Day 1 to Day 3 but increased again on Day 5. By contrast, all the other treatments led to a steady increase of luciferase expression in the growing tumours.

No apparent signs of toxicity or animal weight loss were observed during the experiment, thus showing the good tolerability of all the treatments by the animals (Fig. 10B).

On the last day of the experiment, 10% of the tumours treated with Tf‐bearing liposomes entrapping plumbagin completely disappeared, while another 10% of tumours showed a partial response (Fig. 10C). Following treatment with control liposomes, 20% of the tumours were regressing, and 20% were stable. However, it should be noted that all the mice treated with this formulation had to be euthanized at Day 6 due to their tumours reaching the maximum allowed size (10 mm), unlike those of Tf‐bearing liposomes. By contrast, all the tumours treated with plumbagin solution, blank liposomes, or left untreated were progressive.

The improved therapeutic efficacy observed following treatment with Tf‐bearing liposomes entrapping plumbagin resulted in an extended survival of the mice by 6 days compared with untreated tumours (Fig. 10D).

## Discussion

The therapeutic potential of plumbagin has been limited so far, due to its poor solubility in water, lack of stability, and low oral bioavailability, which hampered its biopharmaceutical applications. Plumbagin also failed to specifically reach tumours at a therapeutic concentration due to its lack of tumour specificity and rapid elimination, with a short biological half‐life.

To overcome these issues, we hypothesized that loading plumbagin into a tumour‐targeted delivery system would enhance the specific delivery of plumbagin to cancer cells and increase the therapeutic efficacy both *in vitro* and *in vivo*, while at the same time reducing the secondary effects to healthy tissues.

Liposomes entrapping plumbagin were successfully prepared by using probe sonication method, as the self‐assembly of lipids into a bilayer is not a spontaneous process and generally requires an input of energy (Gregoriadis, 2006). Transferrin was conjugated to the control liposomes using the thiol–maleimide “click” reaction, which is one of the most widely used thiol‐based bioconjugation techniques for grafting delivery systems with peptides, proteins, or antibodies, due to its high selectivity, rapid reaction (without heat or catalyst), and compatibility with aqueous condition (Stenzel, 2013; Ponte et al., 2016). Our results indicated high level of transferrin conjugation efficiency (50.7 ± 0.5% of the initial Tf added), which was similar to our previous conjugation rate of around 50% obtained when using dimethylsuberimidate as a crosslinking agent (Dufès et al., 2000) or as reported by Lopalco et al. (2018) when conjugating transferrin to dopamine‐loaded liposomes using ethyl(dimethylaminopropyl)carbodiimide coupling reagent.

Plumbagin was highly entrapped in both Tf‐bearing and control liposomes (about 80% entrapment). This was higher than that previously reported when entrapping plumbagin in niosomes (about 52% entrapment) (Naresh et al., 1996) or in PEGylated liposomes made of phosphatidylcholine, cholesterol, and DSPE‐mPEG2000 at a molar ratio of 9:3:0.5 (66% entrapment) (Kumar et al., 2011).

Tf‐bearing and control liposomes loaded with plumbagin displayed small sizes (less than 120 nm), which was similar to that previously reported when entrapping plumbagin in PEGylated liposomes, with a mean size of 115 ± 7 nm (Kumar et al., 2011), or when entrapping plumbagin in folic acid‐conjugated d‐α‐tocopheryl polyethylene glycol 1000 succinate nanomicelles, with a mean size of 128 ± 1 nm (Pawar et al., 2016). As the cut‐off size for extravasation has been found to be 400 nm for most tumours (Yuan et al., 1995), these liposomes have the required sizes to be taken up by the transferrin receptor‐expressing cancer cells.

Tf‐bearing liposomes were bearing negative surface charges, lower than that of control liposomes, which is most likely due to the negative charge of thiolated transferrin (−22.1 ± 1.4 mV). Similar reduction of zeta potential upon transferrin conjugation to delivery systems was observed in our previous studies (Fu et al., 2011; Lemarié et al., 2013; Karim et al., 2017), as well as in previous studies by Kircheis et al. (2001). These negative surface charges would reduce the risk of having electrostatic interactions between the liposomes and the negatively charged serum proteins and cell membrane, therefore resulting in a prolonged blood circulation and decrease of the nonspecific uptake of liposomes by healthy cells. Moreover, vesicles with zeta potential values of ±10 to 20 mV, which is the case for the liposomes formulated in this study, are considered to be relatively stable (Bhattacharjee, 2016).

Tf‐bearing and control liposomes were stable when stored at low temperature, with minimal changes in size and zeta potential, unlike blank liposomes, whose size significantly increased over time. This may be attributed to the presence of plumbagin in the lipid bilayer of the liposomes, thus increasing their rigidity while maintaining their negative surface charge and preventing liposome agglomeration. A similar observation was recently reported by Tsermentseli et al. (2018) regarding the entrapment of shikoni, another natural naphthoquinone compound, in PEGylated liposomes made of DOPC, DSPG, and DSPE‐mPEG2K. The authors reported that the drug‐loaded liposomes also displayed a higher size and zeta potential stability than that of empty liposomes when stored over 28 days at 4°C.

Plumbagin liposomes showed a sustained release of the entrapped drug within 10 h. A decrease in the percentage cumulative release of plumbagin (observed from 4 h for the plumbagin solution and from 10 h for the targeted and control liposomes) can be explained by the fact that plumbagin can spontaneously evaporate once released from the liposomes and in solution. However, the release of plumbagin from the Tf‐bearing liposomes was faster than expected and not pH‐optimal yet. This formulation should therefore be further optimized to prolong drug release as well as accomplish pH‐triggered drug release only extracellularly in the mildly acidic tumour tissues and intracellularly in the more acidic endosomes following cellular internalization. The release of plumbagin from transferrin‐bearing liposomes followed a similar trend as previously described from plumbagin‐loaded liposomes (made of phosphatidylcholine and cholesterol at a 9:1 molar ratio) with 100% cumulative drug release being observed within 12 h at pH 7.4 (Kumar et al., 2011).

*In vitro*, cellular uptake studies demonstrated that the conjugation of transferrin to liposomes significantly increased plumbagin uptake in comparison with control liposomes and plumbagin solution on the three tested cell lines. A similar result was obtained when replacing plumbagin with coumarin‐6 as a lipophilic fluorescent drug model. These results were in agreement with our previous data that showed that the use of transferrin as a targeting ligand on Solulan‐based vesicles improved the uptake of the drugs tocotrienol and epigallocatechin gallate by at least 1.5‐fold for the three tested cell lines (Fu et al., 2009; Lemarié et al., 2013). They were also in line with another study that reported that the cellular uptake of resveratrol in U87MG human glioblastoma cells was increased following treatment with Tf‐bearing liposomes compared with control liposomes (Jhaveri et al., 2018).

The cellular uptake of Tf‐bearing liposomes was partially inhibited by chlorpromazine and filipin, while control liposomes were partially inhibited only by chlorpromazine. These results confirm the involvement of clathrin‐mediated endocytosis, which is the main mechanism of nanomedicine internalization (Alshehri et al., 2018) and caveolae‐mediated endocytosis in the internalization of Tf‐bearing liposomes. The decrease of the drug uptake by the cells was similar to the 20% decrease already reported for Tf/TAT‐liposomes containing doxorubicin in B16 cells, following pretreatment with chlorpromazine (20 μg/ml for 2 h) (Yuan et al., 2016).

Although clathrin‐mediated endocytosis pathway mainly participated in the uptake of both Tf‐bearing and control liposomes, it should be noted that the conjugation of transferrin to the surface of control liposomes also partially changed the main uptake pathway from clathrin‐mediated endocytosis to caveolae‐mediated endocytosis. This could have a significant impact on the therapeutic efficacy of plumbagin in Tf‐bearing liposomes, as the caveosome is a neutral pH endocytic compartment, thus partially avoiding the eventual degradation of the drug by the acidic internal pH of the endosomes and lysosomes that may occur in the clathrin‐mediated endocytosis pathway (Xiang et al., 2012). The use of Tf‐bearing liposomes therefore led to a significant increase of plumbagin uptake by B16‐F10 cells overexpressing Tf receptors. Free plumbagin enters the cells by passive diffusion, while liposomes are taken up by endocytosis, a slower but highly specific process.

The conjugation of transferrin to liposomes increased the antiproliferative activity of plumbagin in the three tested cancer cell lines. These results correlated with the improved cellular uptake of the drug following treatment with the targeted liposomes. Although the highest plumbagin uptake was found in T98G cells after treatment with plumbagin loaded in Tf‐bearing liposomes, improved antiproliferative activities were found in B16‐F10 and A431 cells, probably because T98G cells are more resistant to plumbagin than the two other cell lines. T98G cell line is a glioblastoma, known to be one of the most malignant and aggressive forms of brain cancer due to its high resistance to chemotherapy (Kriel et al., 2018). Glioblastomas have recently been reported to be resistant to the alkylating agent temozolomide (Munoz et al., 2014) and may also be resistant to the alkylating properties of plumbagin (Klotz et al., 2014), therefore limiting its therapeutic efficacy on T98G cells. Our results were in accordance with previously published works. Duraipandy and colleagues have demonstrated that silver caged nanoparticles entrapping plumbagin at a concentration of 2.5 μM (0.47 μg/mL) were able to reduce the cell viability of A431 cells by 80%, while plumbagin solution at the same concentration reduced cell viability by only 20% (Duraipandy et al., 2014). In another work, micelles containing plumbagin were shown to improve its in vitro antiproliferative activity on MCF‐7 cells by 2.1‐fold compared with the drug solution (Bothiraja et al., 2013). Another targeted formulation of plumbagin loaded in aptamer‐targeted PLGA‐PEG nanoparticles was reported to enhance the cytotoxicity of the formulation by two‐fold compared with nontargeted nanoparticles (Pan et al., 2017), in line with our results. However, the IC_50_ of their targeted formulation was 4.78 ± 0.83 μM (0.89 μg/mL), which was higher than what obtained in our experiments on A431 and B16‐F10 cells.

The entrapment of plumbagin in Tf‐bearing liposomes also increased apoptosis in the three tested cancer cell lines, unlike drug solution. This result correlated well with those obtained from the antiproliferative assay, showing that Tf‐bearing liposomes exhibited the highest antiproliferative effect on B16‐F10 cells followed by A431 and T98G cells. Our results were in agreement with previous reports which demonstrated that the treatment with plumbagin entrapped in silver nanocages led to the apoptosis of A431 cells (Duraipandy et al., 2014) and HeLa cells (Appadurai & Rathinasamy, 2015), unlike free plumbagin.

*In vivo*, we demonstrated that the intravenous administration of plumbagin entrapped in Tf‐bearing liposomes led to complete tumour eradication for 10% of B16‐F10 tumours and tumour regression for 10% of these tumours. To our knowledge, it is the first time that the intravenous administration of plumbagin entrapped in a tumour‐targeted delivery system to mice bearing tumours was able to lead to tumour regression and even complete tumour suppression in some cases. Other studies have previously demonstrated the ability of plumbagin entrapped in various delivery systems to slow down the growth of tumours, rather than the tumour regression or suppression observed in some instances in our experiments. The intravenous administration of plumbagin loaded in niosomes (3–6 mg/kg) (Naresh et al., 1996) or in PEGylated liposomes (2 mg/kg) (Kumar et al., 2011) has been reported to slowdown the growth of sarcoma‐180 and B16F1 melanoma model in mice, compared with that observed with the drug solution. Similar therapeutic effects were also observed when changing the route of administration of the delivery systems loaded with plumbagin. The subcutaneous injection of plumbagin entrapped in PLGA microspheres (10 mg/kg) to BALB/C mice resulted in a significant decrease in tumour growth volume of sarcoma‐180 tumours compared with free plumbagin (volume‐doubling times respectively of 14.3 ± 1.5 days and 7.2 ± 0.9 days) (Singh et al., 1996). In another study, the intramuscular administration of plumbagin entrapped in chitosan‐based microspheres (6 mg/kg) to C57BL/6J mice increased the animals' lifespan by 30% compared with free plumbagin (which increased the lifespan by 20%) (Rayabandla et al., 2010).

The most striking effects of the tumour‐targeted liposomes were the induction of tumour regression within 1 day after treatment and the disappearance of the tumours for some animals of this treatment group within 10 days of treatment. In addition, these effects occurred using doses of 2 mg/kg, lower than reported in most other studies. This therapeutic system was able to act on subcutaneous implanted tumours after systemic administration and should therefore have the potential to target multiple metastatic nodules disseminated throughout the body. This therapeutic effect was promising, although short‐lived, and strongly encourages the further improvement of these extremely safe delivery systems, by using a higher dose, slowing the release rate of the drug from the liposomes, increasing the frequency of the treatment (from every 2 days to every day), and extending the length of the treatment, which should hopefully lead to an optimized therapeutic effect.

## Conclusion

In this study, we have demonstrated for the first time that a novel formulation of plumbagin entrapped in a tumour‐targeted delivery system can lead to tumour regression following intravenous administration.

The loading of plumbagin in transferrin‐bearing liposomes significantly enhanced plumbagin uptake by cancer cells, resulting in an improvement of the antiproliferative (by up to 4.3‐fold) and apoptosis efficacies (by up to 5.5‐fold) compared with the drug solution.

*In vivo*, the intravenous injection of Tf‐bearing liposomes entrapping plumbagin led to tumour suppression for 10% of B16‐F10 tumours and tumour regression for a further 10% of the tumours. By contrast, all the tumours treated with plumbagin solution or left untreated were progressive. The animals did not show any signs of toxicity.

These studies provide a proof of principle that the entrapment of plumbagin in a tumour‐targeted delivery system is a highly promising strategy for cancer treatment and should be further investigated to optimize its anticancer therapeutic efficacy.

## Conflict of Interest

The authors have no conflict of interest to declare.
